# Influence of the Duty Cycle of Pulse Electrodeposition-Coated Ni-Al_2_O_3_ Nanocomposites on Surface Roughness Properties

**DOI:** 10.3390/ma16062192

**Published:** 2023-03-09

**Authors:** Aashish John, Adil Saeed, Zulfiqar Ahmad Khan

**Affiliations:** NanoCorr, Energy & Modelling (NCEM) Research Group, Department of Design & Engineering, Bournemouth University, Talbot Campus, Dorset BH12 5BB, UK

**Keywords:** pulse electrodeposition coating, nanocomposites, surface roughness, Ni-Al_2_O_3_

## Abstract

In this study, the viability of duty cycle variation was explored as a potential method to improve the mechanical and surface roughness properties of Ni-Al_2_O_3_ nanocoatings through pulse electrodeposition. The areal and surface roughness properties of nanocomposite pulse electrodeposition-coated materials with varying duty cycles from 20% to 100% was studied with the analysis of bearing area curves and power spectral densities. Results demonstrate that with decrease in duty cycle, there was an enhancement in aerial roughness properties from 0.348 to 0.195 µm and surface roughness properties from 0.779 to 0.245 µm. The change in surface roughness was due to grain size variation, resulting from the varying time intervals during pulse coatings. This increase in grain size with the change in duty cycle was confirmed with the scanning electron microscope. In addition, an increase in grain size from 0.32 to 0.92 µm with an increase in duty cycle resulted in a decrease in nanohardness from 4.21 to 3.07 GPa. This work will provide a novel method for obtaining Ni-Al_2_O_3_ nanocomposite coatings with improved surface roughness and hardness properties for wider industrial applications.

## 1. Introduction

Within the last decade, pulse electrodeposition coatings were considered as a simple, economic, and viable methodology for producing metal matrix composite (MMC) coatings exhibiting high mechanical properties, tribological properties and corrosion-resistant properties [1,2,3,4,5,6]. Pulse electrodeposition is an augmentation of electrodeposition coating, wherein various physical parameters including peak current density, duty cycle, frequency, pH and bath composition of electrolytes can be precisely controlled for obtaining remarkable surface coatings [7,8,9,10]. Hard particles dispersed with pulse electrodeposition methods for developing nanocomposite coatings and their resulting enhanced properties, have earned wide acceptance in chemical, mechanical and electronic industries [3,11,12,13]. The advantageous properties of pulse electrodeposition coating such as low cost, easy design, reduced grain size, high production rates and fewer technological barriers results in the easy conversion from the laboratory state to the industrial scale [7,14,15].

Nickel-based nanocomposites exhibit outstanding properties and are therefore widely used in various petrochemical, mechanical, electromechanical and tribological applications. It is widely known that supplementing ceramic nanoparticles such as Al_2_O_3_, SiC, ZrO_2_, MnO, ZrO, and TiN further improves coating surface characteristics. It was reported that the hardness and tribological properties of Ni-Al_2_O_3_ composite coatings, developed through pulse electrodeposition techniques, are affected by both frequency and duty cycle [16]. It was noticed that a relatively low frequency and a lower duty cycle result in enhanced tribological properties including hardness. It was added that pulse electrodeposition properties can have an influence on hardness and wear characteristics of coatings without any alterations in bath composition. Jegan et al. [17] employed the Watt solution for Ni-Al_2_O_3_ nanocomposite coatings by changing the duty cycle, current density and frequency during deposition of coatings. It was reported that the duty cycle was a predominant factor which influences the hardness of the specimen. Chen et al. [18] varied the frequency of pulse electrodeposition coating for Ni-Al_2_O_3_ and reported that an increase in frequency resulted in a decrease in the hardness of composite coatings. Steinbach et al. [19] reported the lower agglomeration of particles during coating for pulsed electrodeposition coating than direct deposition coating. In addition, the smaller the particles used for coating, the more effective it will be to pin grain boundaries, which will improve the hardness of the surface. Ma et al. [20] fabricated Ni-Al_2_O_3_ coatings using the ultrasonic-assisted electrodeposition method. It was observed that ultrasonic power affected the number of particles which had been incorporated on the surface in addition to their surface roughness. The increase in the ultrasonic power resulted in a change in the hardness of the coated surface. The impact of current density on the pulse electrodeposition coating was studied by Gul et al. [21] with Al_2_O_3_ nanoparticles embedded with Ni. An increase in Al_2_O_3_ deposition was observed with increasing current density, which resulted in an increase in microhardness. In the ON state, particles will be attached to the surface due to the current provided; and in the OFF time, particles which are loosely adsorbed will be detached. In the OFF state, loosely attached agglomerated particles will fall back to the electrolyte. With an increase in current density, particles attached on to cathodic surface increase. It was concluded that microhardness enhancement resulted from a decrease in the grain size and increasing current density. Further, it was noted that pulsed electrodeposition coating had improved properties compared to direct electrodeposition coating in all the current densities.

The properties of pulse electrodeposited coatings can easily be altered with various parameters, for example current density, duty cycle, deposition duration or time, and pH of the electrolytic solution. By changing time period at which the pulses are imposed, the duty cycle of the coating can be varied, which will change the duty cycle of the coating phenomenon. The relation between the time intervals and the duty cycle is given as

$$\gamma =\frac{T_{\mathrm{ON}}}{T_{\mathrm{ON}}+T_{\mathrm{OFF}}}$$

where γ is the duty cycle, T_ON_ is the time when the pulses are imposed and T_OFF_ is the relaxation time. Yang et al. [22] observed that with the surge in the duty cycle, the grain structure became coarser and there was a decrease in hardness and particle incorporation. Similar observations were recorded by Lajevardi et al. [23] during the coating of Ni-TiO_2_. A decrease in microhardness and TiO_2_ deposition was observed with an increase in duty cycle. However, researchers have only focussed on the effects of electrodeposition factors on the morphology, microstructure, hardness, corrosion and anti-wear properties and there has not been much focus on surface roughness properties.

The roughness parameters of a surface can be calculated by two methods: two dimensional (2D) or three dimensional (3D). The majority of engineering and scientific investigations have used 2D roughness analysis. Three-dimensional surface roughness, however, has become more important recently [24,25]. The 2D parameters include Ra, Rq, Rt, Rpm, Rvm, and Rz. Ra represents the arithmetic average height and this or the centre line average is the frequently used roughness parameter for quality purposes. Ra represents the average absolute deviation from the profile mean height. However, Ra cannot be deemed for the roughness, as it shows only the average of peaks and valleys. Rq is the root mean square deviation from the profile mean line, which is more sensitive than Ra. Rq can be more precise than Ra within the context of the roughness of surfaces. The interdistance of highest peak and lowest valley over the sampling line is denoted by Rt. Rpm and Rvm embodies the mean of maximum peaks and valleys in sampling length. Rz is a ten-point average or, in other words, it is calculated by averaging the five highest peaks and the five lowest valleys over the sampling distance. Peaks are a subset of summits, which are places that are higher than their eight closest neighbours. A peak height must be greater than 5% of the surface’s ten-point height in order for it to be considered a summit. All these are parameters for the 2D plane. The 3D surface roughness parameters include Sa, Sq, St, Spm, Svm, and Sz. The terms are synonymous with that of 2D roughness parameters. Development of Ni-Al_2_O_3_ nanocoatings was achieved in this study by varying duty cycle parameters between 20% and 100%. A modified Watts bath was employed to contain pulse electrodeposition coating electrolyte. The microstructural properties of the coated surface were evaluated using an SEM and EDS for the elemental analysis. Although several studies were conducted to assess both the tribological and mechanical properties of Ni-Al_2_O_3_ pulse electrodeposition coating, a comprehensive study on the roughness parameters has yet to be conducted. In this work, an extensive analysis of the surface roughness properties was performed for the coating surface at various duty cycles from 20% to 100%. The nanohardness of the coated surface was studied using a nanoindenter.

## 2. Materials and Methods

This study shows to how pulse electrodeposition was employed as a coating technique. EN1A steel was employed as the cathode while pure Nickel was employed as the anode. Considering the adhesive properties, availability, and cost, EN1A was the substrate. The cathode was produced as a circular shape with a 30 mm diameter and a 3.5 mm height or thickness, so that it was disc shaped. Nickel, the anode, was rectangular with a thickness of 2 mm. The samples were polished with grit papers of 220, 600, 800, and 1200 and roughness was set below 0.05 µm. After that, samples were conditioned by using acetone contained in an ultrasonic agitator for five minutes to remove any surface contaminants.

**Figure 1:**
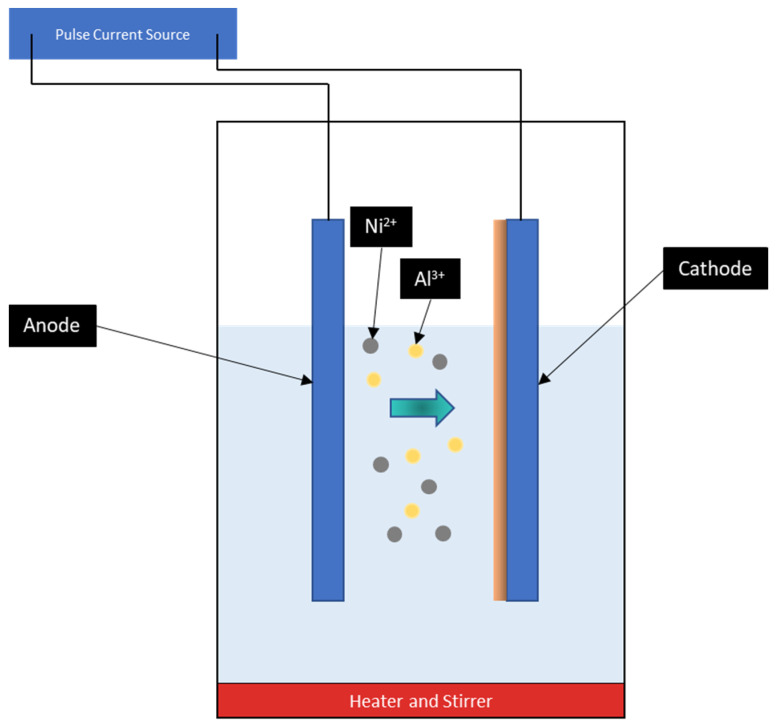
Schematic diagram of pulse electrodeposition coating.

A modified Watts solution was used as the electrolytic bath (Figure 1). The electrolytic chemical composition of the modified Watt solution with their properties is shown in Table 1. Al_2_O_3_ with a particle size less than 50 nm (Sigma-Aldrich, Gillingham, UK) was the reinforcing material used. The constituents were magnetically stirred for 24 h and an additional ultrasonic agitation at a frequency of 10 kHz was provided for 4 h. Appropriate blending and dispersion of nanoparticles in their respective electrolytes were established by magnetic agitation and ultrasonic stirring. The electrolytic solution is heated to 60 °C in the final phase. The detailed test conditions are provided in Table 2.

A pulse power generator was used for the pulse electrodeposition coating technique. For pulse electrodeposition coating, the duty cycle was the only varying factor. All other parameters—current density, frequency, pH of electrolyte, stir speed and electrolyte temperature—were kept constant. The range of the duty cycle and various other parameters were established from previous research and trials [26,27,28,29,30,31]. An increase in the duty cycle from 20% to 100% was achieved incrementally by 20%. Frequency was fixed at 10 kHz and pH was maintained at 4.2 ± 0.2 and 3 A/dm^2^ constant current density. Pulse electrodeposition was conducted at a solution temperature of 60 °C with continuous magnetic stirring and ultrasonic agitation throughout the process. The coating process was set to 1 h. After coating, distilled water was used to condition samples followed by acetone conditioning in an ultrasonic bath to remove all chemicals attached to the surface. Each coating was performed 3 times to determine the repeatability of the coating.

The samples were studied under a non-contact 3D optical profilometer and various parameters including Ra, Rq, Rt, Sa, Sq, St, kurtosis, and skewness were analysed with the power spectral density (PSD) and bearing area curve (BAC). The nanohardness of surfaces was obtained by utilising a nanoindenter. A Berkovich, 3-faced pyramidal, indenter was used for this study. A total of 15 indentations were conducted on the coatings and the results were analysed.

The microstructure of the coating was obtained with using a Scanning Electron Microscope (JEOL). The grain size of coated materials was obtained with ImageJ, an open license software.

## 3. Results and Discussions

### 3.1. Surface Morphology

**Figure 2:**
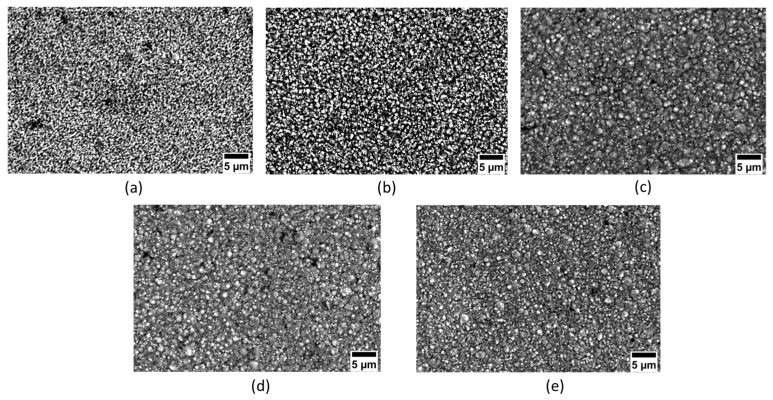
Scanning electron microscope images of coated samples at varying duty cycles: (**a**) 20%, (**b**) 40%, (**c**) 60%, (**d**) 80% and (**e**) 100%.

Figure 2 shows the surface morphology of the samples at various duty cycles. An increase in grain size is seen with a rise in the duty cycle. Electrodeposition coating with a duty cycle of 20% exhibited the smoothest surface. As the duty cycle increases, the size of the particles increased. The change in grain size can be confirmed by the free energy of nucleation for new grains [22]. During T_ON_ of pulse electrodeposition coating, the nanoparticles present in the electrolyte solution will be pulled towards the cathode surface and will be embedded in them. During T_OFF_, while the current supply is cut out, loosely embedded and agglomerated particles will be detached from the cathode surface and will return to the electrolytic solution. This process will be repeated henceforth. With a decrease in the duty cycle, the T_OFF_ time will be increased, as a result of which agglomeration of the particles will decrease, and grains will become finer. Once the duty cycle is increased (a decrease in T_OFF_ and an increase in T_ON_), particle embedding will increase, resulting in more agglomerated particles. This results in a decrease in the nucleation rate and causes an increase in grain growth [32].

### 3.2. Roughness Analysis

**Figure 3:**
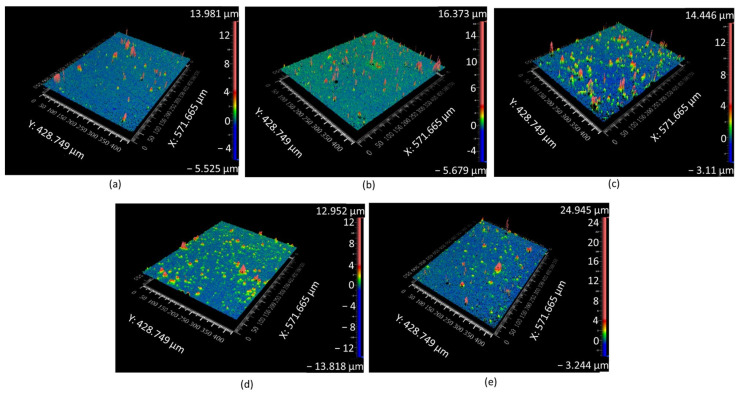
Profilometer images of the duty cycle: (**a**) 20%, (**b**) 40%, (**c**) 60%, (**d**) 80% and (**e**) 100%.

**Figure 4:**
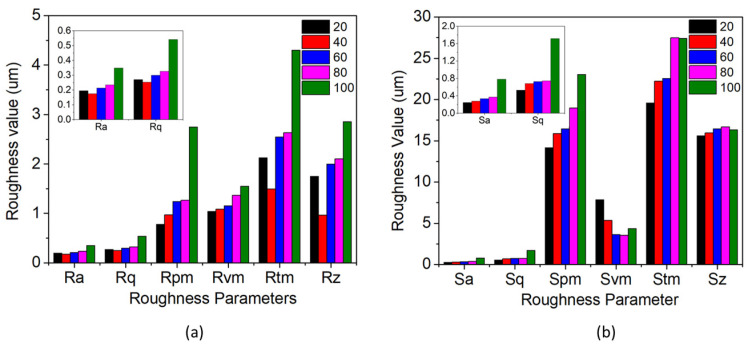
(**a**) Areal roughness parameters and (**b**) surface roughness parameters for different duty cycles.

Figure 3 shows profilometer images of the samples at various duty cycles. The areal roughness parameters and surface roughness parameters of all samples were analysed. Various areal roughness parameters including the Ra, Rq, Rz, Rpm, Rvm, Rt and Rz of the samples at varying duty cycles is shown in Table 3 and Figure 4a. At a duty cycle of 20%, the Ra value was 0.195 µm. Ra increased with an increase in the duty cycle. The Ra value was 0.175, 0.214, 0.234 and 0.348 µm when the duty cycle was 40%, 60%, 80% and 100%, respectively. In order to obtain more accurate areal roughness parameters, Rq was analysed. Rq values also increased -0.271, 0.253, 0.299, 0.326, 0.541 µm as the duty cycle increased from 20%, 40%, 60%, 80% to 100%, respectively.

The average peaks and valleys of the areal surface were also analysed. The average areal peak height of the samples increased when the duty cycle increased. The 100% duty cycle samples had the highest peak of 2.748 µm and decreased when the duty cycle decreased. The peak height of 80%, 60%, 40%, and 20% was 1.267, 1.244, 0.972, and 0.781 µm, respectively. Similarly, the areal valley height of the samples was analysed. The valley height increased from −1.043 to −1.084 µm when the duty cycle increased from 20% to 40%. Thereafter, a slight decrease in height of −1.155 µm was observed at a 60% duty cycle, which further increased to −1.368 and −1.550 µm at 80% and 100%, respectively. The Rt was observed to increase when the duty cycle increased from 2.125 to 4.299 µm at 20% and 100% duty cycles, respectively.

The Rz values were analysed to achieve comprehensive knowledge of the average ten-point height of the profile in the sampling length. At a 20% duty cycle, the Rz was 0.752 µm, which increased to 0.967, 1.998, 2.107, and 2.857 µm for duty cycles of 40%, 60%, 80%, and 100%. Therefore, it is reasonable to conclude that, with an increase in the duty cycle, the average maximum height difference in the surface increases.

Surface roughness parameters were analysed to understand roughness characteristics in the entire surface and are shown in Table 4 and Figure 4b. The surface roughness parameter calculates the roughness of the whole surface, whereas the areal roughness parameter will calculate the roughness of the singular plane. The Sa value of 20% was 0.245 µm. The Sa values increased correspondingly with the duty cycle. For 40%, 60%, 80% and 100%, the values were 0.277, 0.334, 0.371, and 0.779 µm, respectively. A similar increase was observed for Sq values of 0.529, 0.682, 0.728, 0.742, and 1.714 µm for DC from 20% to 100%, respectively. The average peak and average valley heights were also noted and had a similar trend to that of the areal roughness parameters increasing height when the duty cycle increased. St and Sz showed a similar trend to that of the aerial roughness properties. The maximum peak height will influence the tribological properties of the surface as the peaks will restrict the complete contact of the counter surface with the material [33]. Similarly, the increase in valley height increases retention of the lubricant, which improves the tribological properties of the surface [33].

The variation in skewness and kurtosis with a change in the duty cycle was also analysed and shown in Table 5. The skewness parameter reacts strongly to isolated deep dips or extreme peaks. In the present pulse electrodeposition coating, all the Ssk values are positive, which implies that the surface is skewed downward relative to the mean line. The lower duty cycle samples, 20%, tends to have more peaks and filled valleys. With the rise in the duty cycle from 20% to 100%, skewness decreases, which implies that the number of peaks in the surface decreases.

The sharpness of the peaks on a surface can be defined as the kurtosis or Sku. Sharper peaks and heavier tails project a positive kurtosis. The same positive kurtosis can be observed in all duty cycle samples, pointing towards the relatively high peaks and broader valleys at the surface. It can also be observed that with a decline in the duty cycle, the kurtosis increases. The positive excess kurtosis value projects the distribution of more peaks and fatter valleys. That is, the distribution shows peaks close to the mean value and valleys, which are extremely frequent compared to the normal distribution. These distributions are also known as leptokurtic or leptokurtotic, and improve the tribological properties by retaining more lubricant on the surface [34]. Positive skewness and kurtosis values greater than 3 show that the samples can have better tribological properties. To substantiate these findings, bearing area curves were explored and analysed. However, the hardness of the coating plays a similar role in tribology and has to be analysed.

### 3.3. Bearing Area Curves

**Figure 5:**
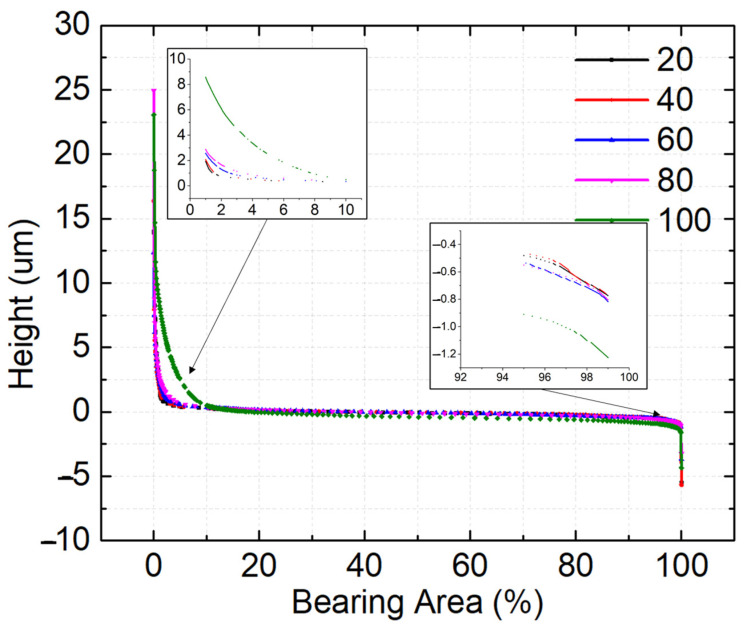
Bearing area curves for different duty cycles.

A broad-spectrum view of the variation in roughness can be obtained using bearing area curves provided in Figure 5. The peak–valley variations and the basic roughness of the samples with different duty cycles can be analysed the bearing area curve (BAC). The percentages of peaks, MR1, and the percentages of valleys, MR2, can be obtained from the BAC. Furthermore, the reduced valley height, Rvk, the reduced peak height, Rpk, and the core or basic roughness, Rk, can be acquired from the BAC.

**Figure 6:**
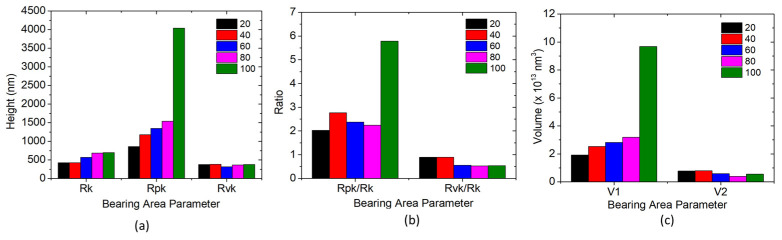
Bearing area parameters for different duty cycles (**a**) Rk, Rpk and Rvk (**b**) Ratio of Rpk/Rk and Rvk/Rk (**c**) Material Ratio.

From Figure 5 and Figure 6a, it can be seen that the number of peaks were at a maximum for samples with a 100% duty cycle. Increasing the duty cycle reduces the number of peaks. The increased Rpk values imply that the surface is composed of a higher number of peaks. The high peaks provide a lower contact area during tribo testing, which will result in increased contact stress. A lower surface contact area contributes to a reduction in friction and wear properties during the running in stage of tribo testing. On the other hand, the decrease in the Rvk values represents the increased number of valleys in the surface below the core surface Rk. With an increasing amount of valley, lubrication retention capacity will increase. The increase in the lubrication retention capacity reduces inter-surface wear and friction values. The ratios of Rpk/Rk and Rvk/Rk are analysed to simplify the process and illustrated in Figure 6b. Rpk/Rk and Rvk/Rk analyses determine whether the surface is dominated by peaks or valleys and the values are shown in Table 6. The Rpk/Rk values dominate the Rvk/Rk for all duty cycles. The Rpk/Rk values increase with an increasing duty cycle −2.03, 2.77, 2.38, 2.24, and 5.79 at 20%,40%, 60%, 80% and 100%, respectively. An increased number of peaks reduce the contact area, which improves the tribological properties of interfaces. Similarly, the Rvk/Rk values decrease when the duty cycle is increased −0.89 for a 20% duty cycle, which decreases to 0.54 for a duty cycle of 100% [33].

V1 and V2 in Figure 6c represent material deposition profile peak area and the lubricant-filled profile valley area, respectively. The V1 value for a 20% duty cycle was 1.92 × 10^13^ nm^3^, which increased to 2.52 × 10^13^ nm^3^ for a duty cycle of 40%. A steep increase in the V1 value was observed at 100%, 9.67 × 10^13^ nm^3^. It can be concluded that the peak volume increased by the amount by which the duty cycle increased. Similar observations were seen in V2 as well. A decrease in V2 is seen when the duty cycle is increased. The V2 was 0.76 × 10^13^ nm^3^ for a 20% duty cycle, which increased to 0.79 × 10^13^ nm^3^ at a 40% duty cycle. A decrease in the value was observed at 60% and 80% duty cycles—0.57 × 10^13^ and 0.39 × 10^13^ nm^3^, which finally increased to 0.54 × 10^13^ nm^3^ for a 100% duty cycle. In lieu of the surface roughness parameters, the hardness values are also expected to be higher for the lower duty cycle samples.

### 3.4. The Power Spectral Density

**Figure 7:**
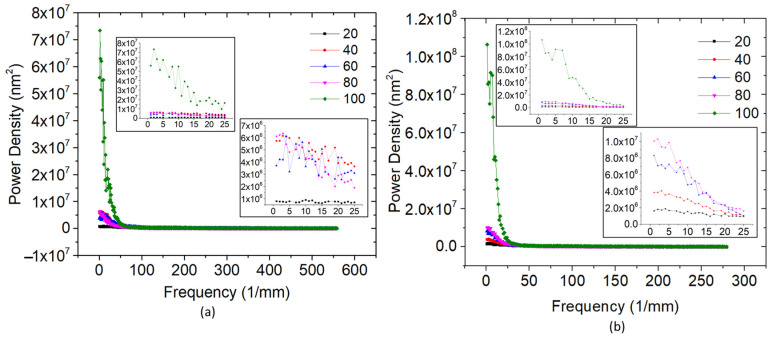
Power spectral analysis for different duty cycle samples in (**a**) *x*-axis and (**b**) *y*-axis.

Figure 7 illustrates plots exhibiting a disparity in power spectral density (PSD) with spatial frequency for duty cycles of 20%, 40%, 60%, 80% and 100% for the *x*-axis and the *y*-axis. Spatially dependent surface profiles of the samples with various duty cycles—20%, 40%, 60%, 80% and 100%—were analysed in the present PSD analysis. A Fourier series decomposes the input surface profile’s roughness frequency (spatial frequency). Each roughness frequency’s power spectral density is recorded and averaged for PSD function calculation. Figure 7 shows the results of these analyses with spatial frequency and power density on *x*-axis and *y*-axis of surface. It is evident from Figure 7a,b that the power density is high for a duty cycle of 100% at a lower frequency range, 0–25/mm. For the same range, 0–25/mm density decreases with a decrease in the duty cycle, with 20% having the least roughness profile variation. In both cases, the amplitude decreases with an increase in spatial frequency. This shows that the dominating surface roughness is from mid-range and lower spatial frequencies. The power spectrum is nearly smooth in all the duty cycles after a spatial frequency of 60/mm. The roughness frequency is independent of the flat portions with the same average roughness values [35].

### 3.5. Nanohardness

**Figure 8:**
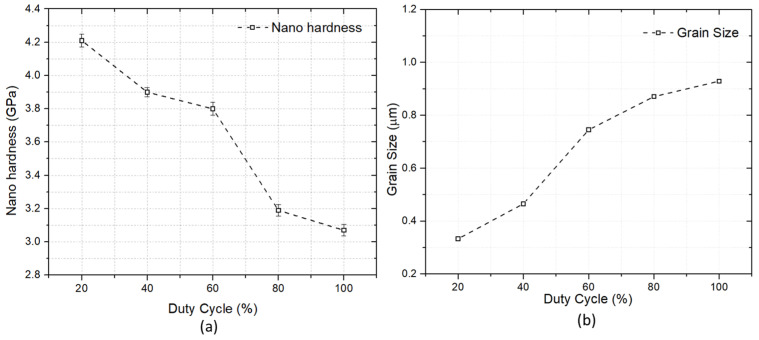
(**a**) Nanoindentation hardness for Ni-Al_2_O_3_ coatings at different duty cycles. (**b**) Grain size of coatings at various duty cycles.

Figure 8 shows the hardness pattern influenced by changes in the duty cycle. The pattern shows that the nanohardness reduced with an increase in the duty cycle. A significant impact was observed in nanohardness with the change in the duty cycle. The maximum nanohardness was observed at a duty cycle of 20% with a 4.21 GPa. For a duty cycle of 40%, 60%, 80% and 100%, the nanohardness was 3.90, 3.86, 3.19 and 3.07 GPa, respectively. Results indicate that the duty cycle has an impact on the hardness of the coating.

The change in hardness can be explained by the Hall–Patch equation, relating hardness and grain size.

$$\mathrm{HV}={\mathrm{HV}}_{o}+\frac{K}{\sqrt{d}}$$

where hardness is denoted by HV, HV_o_ and K are both constants, and d represents the grain size. The coating hardness is affected by the grain size. The increase in grain size with an increasing duty cycle has already been observed earlier (Figure 2) and the grain size is calculated using open software, ImageJ. The variation in the grain size with the change in the duty cycle is plotted in Figure 8b. The decrease in hardness with an increase in the duty cycle can be attributed to the Hall–Patch relationship. The higher the nanohardness for a duty cycle of 20%, the better the wear properties.

## 4. Conclusions

In this study, electrodeposition with varying duty cycles from 20% to 100% was employed to develop Ni-Al_2_O_3_ nanocomposites. Various areal and surface roughness properties of nanocomposite coatings were analysed. It was observed that the roughness values increased when the duty cycle increased. Both the skewness and kurtosis values of the surfaces were analysed and it was observed that the skewness decreased with an increased duty cycle. Similarly, with an increase in the duty cycle, kurtosis decreased. However, the kurtosis for all duty cycles was greater than 3 and all had a positive skewness. A positive skewness and a higher kurtosis reduces friction, so the 20% duty cycle has the best wear properties. The nanohardness of the samples was analysed and it was observed that a lower duty cycle had a higher hardness, which improves the tribological properties of the surface. The decrease in nanohardness at a higher duty cycle was due to the increase in the grain size resulting from a shorter OFF time.

## Figures and Tables

**Table 1:** Electrolytic composition.

Chemical Composition	Quantity (g/L)	Function
Nickel Sulphate Hexahydrate (NiSO_4_·6H_2_O)	265	Nickel Source
Nickel (II) Chloride (NiCl_2_·6H_2_O)	48	Conductivity and Nickel Source
Boric Acid (H_3_BO_3_)	31	Buffering agent and Stabilizer
Aluminium Oxide (Al_2_O_3_)	10	Reinforcing agent

**Table 2:** Test conditions for coatings.

	Parameter 1	Parameter 2	Parameter 3	Parameter 4	Parameter 5
Current Density (A/dm^2^)	3				
Duty Cycle (%)	20	40	60	80	100
Frequency (kHz)	10				
Time (min)	60				
pH	4.2 ± 0.2				
Temperature (°C)	60 ± 5				
Anode	Nickel Plate				
Cathode	EN1A				

**Table 3:** Areal roughness of samples with different duty cycles.

Areal Roughness Parameters (µm)						
Duty Cycle	Ra	Rq	Rpm	Rvm	Rtm	Rz
20	0.195	0.271	0.781	1.043	2.125	0.752
40	0.175	0.253	0.972	1.084	1.495	0.967
60	0.214	0.299	1.244	1.155	2.549	1.998
80	0.234	0.326	1.267	1.368	2.635	2.107
100	0.348	0.541	2.748	1.550	4.299	2.857

**Table 4:** Surface roughness of samples with different duty cycles.

Surface Roughness Parameters (µm)						
Duty Cycle	Sa	Sq	Spm	Svm	Stm	Sz
20	0.245	0.529	14.161	7.830	19.560	15.612
40	0.277	0.682	15.865	5.345	22.212	15.974
60	0.334	0.728	16.427	3.631	22.559	16.436
80	0.371	0.742	18.948	3.531	27.479	16.687
100	0.779	1.714	23.046	4.350	27.396	16.339

**Table 5:** Skewness and kurtosis for various duty cycles.

Skewness and Kurtosis		
Duty Cycle	Ssk	Sku
20	8.404	124.368
40	8.566	118.789
60	7.171	82.631
80	7.365	51.362
100	5.698	46.365

**Table 6:** Bearing area parameters for different duty cycles.

Bearing Area Parameters									
Duty Cycle	Rk (nm)	Rpk (nm)	Rvk (nm)	Rpk/Rk	Rvk/Rk	Mr1 (%)	Mr2 (%)	V1 (×10^13^ nm^3^)	V2 (×10^13^ nm^3^)
20	422.495	858.300	376.515	2.032	0.891	16.74	82.885	1.920	0.769
40	423.675	1174.255	380.045	2.772	0.897	17.97	82.910	2.525	0.7865
60	567.250	1348.270	314.545	2.377	0.555	18.49	84.415	2.820	0.576
80	685.720	1535.510	364.475	2.239	0.532	19.215	90.980	3.185	0.393
100	697.960	4040.490	376.69	5.789	0.540	19.73	92.080	9.670	0.545
